# Acetabular Bone Defect in Total Hip Arthroplasty for Crowe II or III Developmental Dysplasia of the Hip: A Finite Element Study

**DOI:** 10.1155/2020/4809013

**Published:** 2020-08-25

**Authors:** Yinqiao Du, Jun Fu, Jingyang Sun, Guoqiang Zhang, Jiying Chen, Ming Ni, Yonggang Zhou

**Affiliations:** Department of Orthopaedics, Chinese People's Liberation Army General Hospital, 28 Fuxing Road, Haidian District, Beijing, China 100853

## Abstract

**Background:**

The purpose of this study was to establish the finite element analysis (FEA) model of acetabular bone defect in Crowe type II or III developmental dysplasia of the hip (DDH), which could evaluate the stability of the acetabular cup with different types of bone defects, different diameters of femoral ceramic heads, and the use of screws and analyze the stress distribution of screws.

**Methods:**

The FEA model was based on the CT scan of a female patient without any acetabular bone defect. The model of acetabular bone defect in total hip arthroplasty for Crowe II or III DDH was made by the increasing superolateral bone defect area of the acetabular cup. Point A was located in the most medial part of the acetabular bone defect. A 52 mm PINNACLE cup with POROCOAT Porous coating was implanted, and two screws (the lengths were 25 mm and 40 mm) were implanted to fix the acetabular cup. The stability of the acetabular cup and the von Mises stress of point A and screws were analyzed by a single-legged stance loading applied in 1948 N (normal working). The different diameters of the femoral ceramic head (28 mm, 32 mm, and 36 mm) were also analyzed.

**Results:**

The von Mises stress of point A was gradually increased with the increasing uncoverage values. When the uncoverage values exceeded 24.5%, the von Mises stress of point A without screws increased significantly, leading to instability of the cup. Screws could effectively reduce the von Mises stress of point A with uncoverage values of more than 24.5%. However, the peak von Mises stress in the screws with the uncoverage values that exceeded 24.5% was considerably increased. The diameter of the femoral ceramic head had no significant effect on the von Mises stress and the stability of the acetabular cup.

**Conclusions:**

We recommend that uncoverage values of less than 24.5% with or without screw is safe for patients with Crowe II or III DDH.

## 1. Introduction

Total hip arthroplasty (THA) is one of the most successful surgeries in the 20^th^ century; a gradually increasing amount of patients with developmental dysplasia of the hip (DDH) received THA to release pain and improve function [1–3]. Despite their surgical complexity, DDH patients had also notably low rates of revision and obtained durable clinical results [2, 4–7]. During THA surgery, an obvious superolateral bone defect has been reported to be frequently observed above the surface of the acetabular cup at the level of the true acetabulum, especially in patients with Crowe II or III DDH [7, 8].

It has been recommended that the acetabular cup uncoverage should not exceed 30% of its overall surface [9]. If the uncoverage was more than 30%, a structural bone grafting may be needed [10–12]. However, all of these decisions were made which depended on the intraoperative judgment of surgeons. There is no previous publication reporting the specific value of uncoverage in THA of DDH patients.

The aim of this current study was to establish a finite element analysis (FEA) model of acetabular bone defect in THA for Crowe II or III DDH, in which the acetabular cup was positioned at the anatomical center of rotation of the hip without grafting. Then, the stability of the acetabular cup with different types of bone defects, different diameters of femoral ceramic heads, the use of screws, and the stress distribution of screws were analyzed.

## 2. Materials and Methods

The FEA model was based on the geometry of a human pelvis, obtained from the CT scan of a female patient (bodyweight: 50 kg) without any acetabular bone defect, who signed an informed consent for this study. The three-dimensional model of the pelvis was restored using Mimics Research 20.0 (Materialise, Belgium). The STL date of the pelvis was used to conduct the reverse engineering reconstruction using Geomagic 2012 (Geomagic, America), which includes segmentation, smoothing, polishing, denoising, and other image processing of the model. Then, this solid model was generated into IGES three-dimensional image. We followed the methods of Fu et al. [13].

According to the actual size of the patient's acetabulum and femur, acetabular and femoral prostheses were assembled using Solidworks 2014 (Dassault, France). The acetabular cup was a 52 mm PINNACLE cup with POROCOAT Porous coating (DePuy, America); cup inclination of 40 degrees and anteversion of 20 degrees were preset using a coordinate system linked with the pelvis. The ceramic liner (DePuy, America) and size 9 LCU femoral prosthesis (Link, Germany) with 28 mm/32 mm/36 mm femoral ceramic head of different diameters (Link, Germany) were implanted. When the diameter of the femoral ceramic head was 36 mm, two screws (the lengths were 25 mm and 40 mm, and the diameter was 6.5 mm) were implanted to fix the acetabular cup.

The solid model was imported into Ansys Workbench 16.2 (Ansys, America), and Boolean operation was performed. Mesh generation was made after setting up all the material properties and interfaces. The mesh size was set as 1 mm using an automatic mesh technique, as validated in a previous study [14]. Element type was chosen as Solid 187. The material properties used in the model are presented in Table 1. The friction coefficient between the cup and the bone was 0.8. And the friction coefficient between the ceramic liner and the femoral ceramic head was 0.06 [15].

**Figure 1:**
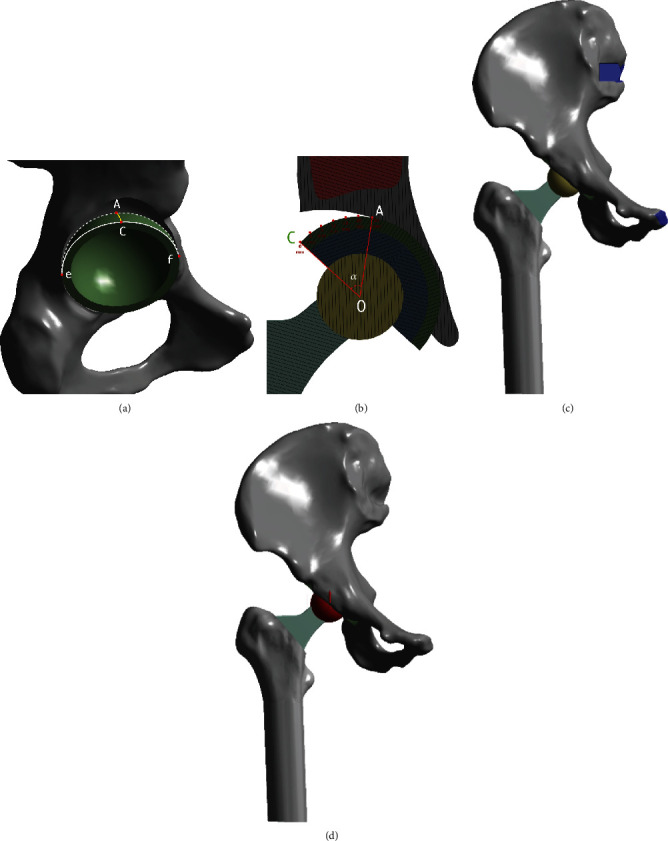
(a) The model of acetabular bone defect in total hip arthroplasty for Crowe II or III DDH. (b) The uncoverage area gradually increased by the length of the arc AC, and the angle *α* denoted the central angle of the uncovered portion above the cup. Angle *α* was formed by the crossing of line OA and OC. (c) Fixed constraint boundary conditions were assumed at the sacroiliac joint and pubic symphysis. (d) Simulated vertical reaction load was applied from the bottom of the femur.

The model of acetabular bone defect in THA for Crowe II or III DDH was made as shown in Figures 1(a) and 1(b).  Figure 1(a) shows the sector defect, which was identified by extending the arc (defined as arc eAf) formed by the bone-prosthesis border within superolateral bone defect to the edge which was defined by arc eCf, and the arc AC was the longest distance from the sector defect [7, 16]. The straight line ef went through the hip center of rotation (HCOR). The surface area of the defect gradually increased by the length of the arc AC, and the angle *α* denoted the central angle of the uncovered portion above the cup as shown in Figure 1(b).

A resultant equivalent load (single-legged stance loading) was applied without taking account of muscles around the hip joint. The peak force measurements for the unilateral hip joint were reported in the majority of literature showing 1948 N for normal working [17, 18]. Fixed constraint boundary conditions were assumed at the sacroiliac joint and pubic symphysis (Figure 1(c)). The femur was constrained in all directions at the middiaphysis, and the simulated vertical reaction load was applied from the bottom of the femur at 1948 N (Figure 1(d)).  Figure 1 shows the general layout used for these FEA models and the details of components, interfaces, load, and constraint boundary. The main analysis was the effect of bone defect, femoral ceramic head, and screw on the stability of the acetabular cup.

## 3. Results

### 3.1. The Effect of Different Bone Defects and Femoral Ceramic Head on the Stability of the Acetabular Cup

**Figure 2:**
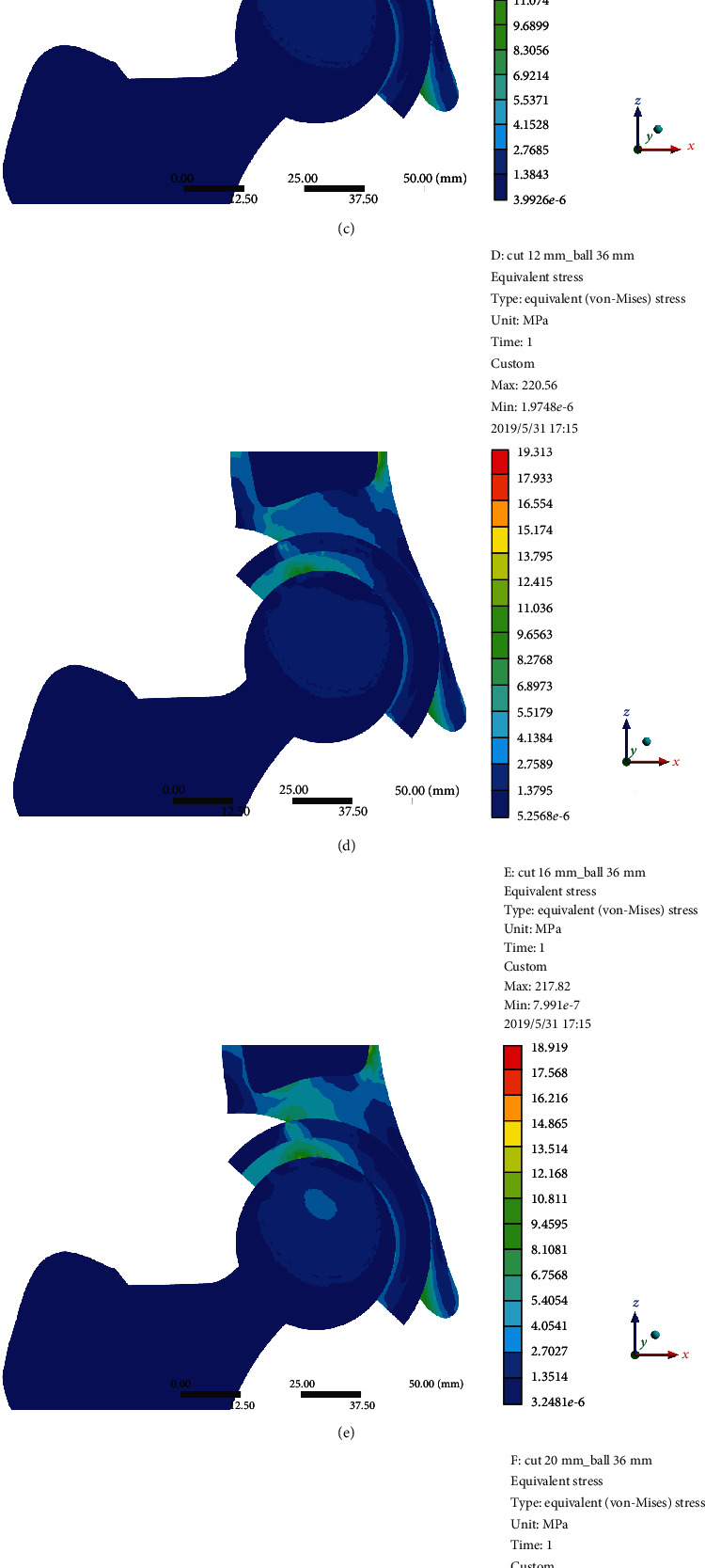
The von Mises stress of point A without screw. (a) The length of arc AC was 0 mm with a 36 mm femoral ceramic head. (b) 4 mm. (c) 8 mm. (d) 12 mm. (e) 16 mm. (f) 20 mm. (g) 24 mm. (h) Relationship between the von Mises stress of point A (MPa) and the length of arc AC with different diameters of the femoral ceramic head.

According to the result of FEA, the diameter of the femoral ceramic head had no significant effect on the von Mises stress of point A and the stability of the acetabular cup. The von Mises stress of point A was gradually increased with the increasing length of the arc AC (uncoverage). When the length of the arc AC exceeded 20 mm (uncoverage > 24.5%, angle *α* > 44.0°), the von Mises stress of point A improved significantly, leading to prosthesis instability (Figure 2).

### 3.2. The Effect of Screws on the Stability of the Acetabular Cup

**Figure 3:**
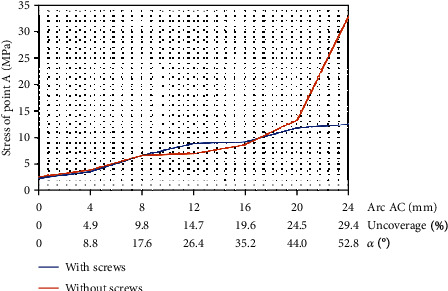
Relationship between the von Mises stress of point A (MPa) and the length of arc AC with and without screw.

When the diameter of the femoral ceramic head was set on 36 mm and two screws were inserted into the acetabular cup, the von Mises stress of point A with two screws was 12.44 MPa (without screw: 32.98 MPa) on the condition that the length of arc AC was 24 mm (uncoverage: 29.4%, angle *α*: 52.8°) and the acetabular cup with two screws had no sign of instability (Figure 3).

### 3.3. Stress Distribution of the Screw for Fixing an Acetabular Cup

**Figure 4:**
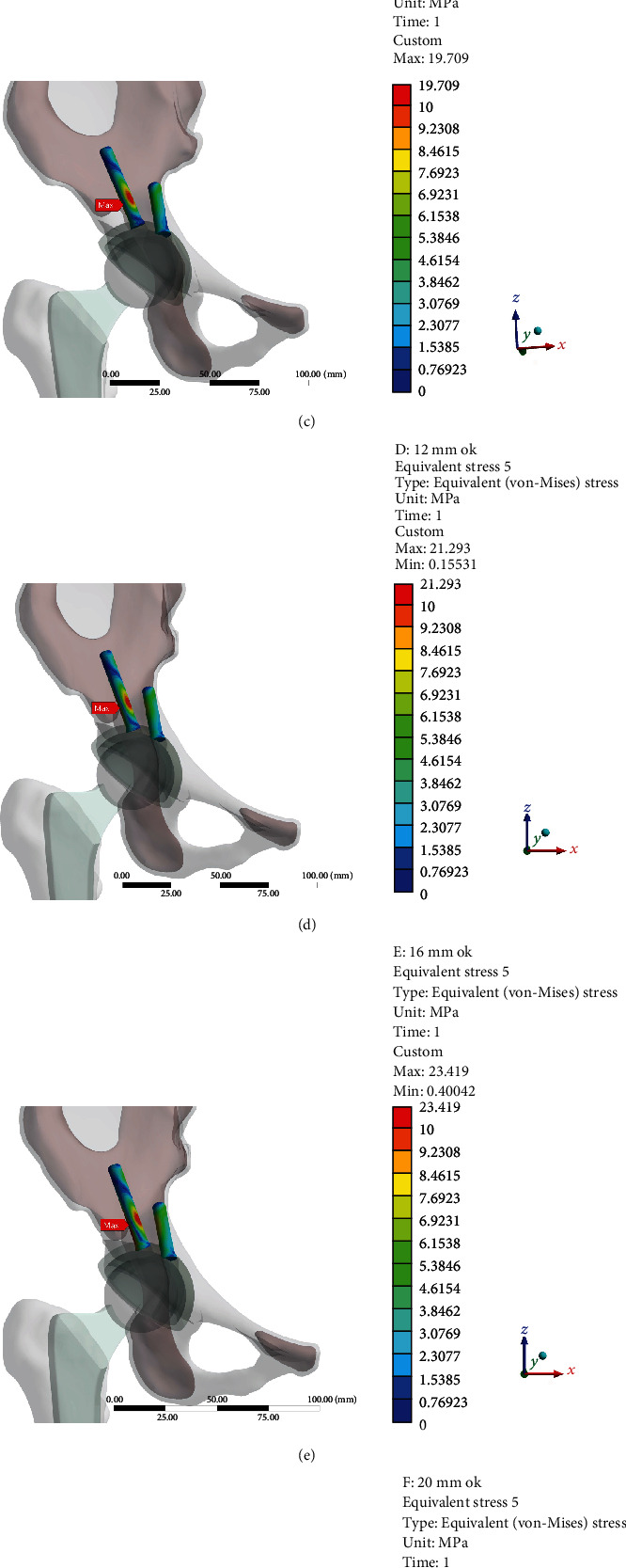
The stress distribution of the screw for fixing an acetabular cup: (a) 0 mm; (b) 4 mm; (c) 8 mm; (d) 12 mm; (e) 16 mm; (f) 20 mm; (g) 24 mm.

The peak von Mises stress of the two screws was located in the upper 1/3 of the two screws, which was 18.83 MPa (0 mm), 19.06 MPa (4 mm), 19.71 MPa (8 mm), 21.29 MPa (12 mm), 23.42 MPa (16 mm), 62.12 MPa (20 mm), and 62.09 MPa (24 mm), respectively. As the he length of the arc AC increased, the peak von Mises was gradually moved down along the screw (Figure 4).

## 4. Discussion

This current study of a three-dimensional FEA model of acetabular bone defect in THA for Crowe II or III DDH was constructed from CT scan date and used to analyze the effect of bone defect, femoral ceramic head, and screws on the stability of the acetabular cup. The results showed that the diameter of the femoral ceramic head had no significant effect on the von Mises stress and the stability of the acetabular cup; the acetabular cup primary stability can be achieved with uncoverage values of less than 24.5% (angle *α*: 44°) without a screw. Screws could effectively reduce the von Mises stress of point A with uncoverage values of more than 24.5%. However, the peak von Mises stress in the screws considerably increased.

In Crowe II or III DDH, the superolateral acetabular deficiency prevents placement of a standard cup to inadequate coverage [16]. Special techniques including high hip center and bone graft may be necessary to address inadequate osseous coverage of the acetabular cup. There is no standard high hip center technique to guide the process of acetabular reconstruction, which leads to the variable clinical outcomes resulting from the high hip center technique. In addition, it remains unclear to what extent the high hip center technique restores the normal hip biomechanics [19]. Structural bone grafting with the acetabular cup at the level of the true acetabulum was another alternative to reconstruct the defect in DDH patients [20–22]. However, when coverage of the cup by the autograft did not exceed 50%, there will be a high risk of failure in the acetabular cup. And the absorption and collapse of structural bone grafting were also other causes for the failure of structural bone grafting. Therefore, the optimal acetabular cup position is at the level of the true acetabulum that restored the HCOR, the limb-length discrepancy (LLD), and muscle tension around the hip.

Xu et al. [7] measured the three-dimensional coverage postoperatively in 35 patients (45 hips) with Crowe II or III DDH, in which the acetabular cup was positioned at the anatomical center of rotation of the hip. Their research results showed that the postoperative three-dimensional coverage was 85.74% and the height of the uncovered (the length of the arc AC in our study) is a useful parameter to determine the three-dimensional coverage during surgery.

Tikhilov et al. [12] utilized a mathematical computer model based on the FEA and the mechanical experiment to estimate the critical values of uncoverage enabling safe primary fixation of the acetabular cup in arthroplasty patients with DDH. According to their results, cup prosthesis primary stability can be achieved with uncoverage values of less than 15-25% without screw fixation and can reach approximately 35% with two-screw fixation. In our study, a possibility of mounting an acetabular cup with uncoverage within 24.5% (the length of arc AC: 20 mm, angle *α*: 44°) was demonstrated. This study was consistent with most previous studies that the minimal acetabular cup uncoverage should not exceed 30% of its surface [9–11, 23].

Tikhilov et al. suggested that if extreme uncoverage values of greater than 35% of the acetabular cup were observed, screw fixation did not improve reliable primary stabilization [12]. In our study, the von Mises stress of point A with two screws was considerably decreased on the condition that the length of arc AC was 20 mm (uncoverage: 24.5%, angle *α*: 44°) compared with that without screw. The use of screws can effectively reduce the stress of point A and improve reliable primary stabilization of the cup. However, the peak von Mises stress in the screws for fixing the acetabular cup with the length of arc AC exceeding 20 mm (uncoverage > 24.5%, angle *α* > 44.0°) was considerably increased.

There are some limitations. This study investigated an ideal model and simulated acetabular bone defect in THA for Crowe II or III DDH which does not necessarily mimic actual THA surgical environments and the real-life stresses. Additionally, the angle *α* may not be accurate on the X-ray given complex acetabular bone defects for Crowe II or III DDH. The intraoperative measuring or CT scan may better guide patients with early weight-bearing exercises. We believe our mode may provide a surgical guidance to surgeons while performing THA for patients with Crowe II or III DDH.

## 5. Conclusions

We recommend that uncoverage values of less than 24.5% (angle *α* < 44°) with or without a screw are safe for patients with Crowe II or III DDH, in which the acetabular cup was positioned on the anatomical center of rotation of the hip. The use of screws can effectively improve the reliable primary stabilization of the cup when the uncoverage values are more than 24.5%.

## Figures and Tables

**Table 1:** Mechanical properties of materials used in FEA mode.

Components	Materials	Elastic modulus (MPa)	Poisson's ratio
Cortical bone	Cortical bone	17300	0.265
Cancellous bone	Cancellous bone	400	0.2
Screws Acetabular component Femoral prosthesis	Titanium alloy	110600	0.326
Ceramic liner Femoral ceramic head	Ceramics	350000	0.22

## Data Availability

The data used to support the findings of this study are included within the article.
